# Case report: Long-term local control of recurrent lymphatic malformations using minocycline sclerotherapy following surgical resection in a dog

**DOI:** 10.3389/fvets.2026.1910356

**Published:** 2026-09-17

**Authors:** Haruto Taki, Toshikazu Sakai, Takahiro Kondo, Daigo Yokoyama, Jumpei Fujiki, Shushi Yamamoto, Shidow Torisu

**Affiliations:** 1 Laboratory of Companion Animal Surgery, School of Veterinary Medicine, Rakuno Gakuen University, Ebetsu, Japan; 2 Laboratory of Veterinary Biochemistry, School of Veterinary Medicine, Rakuno Gakuen University, Ebetsu, Japan

**Keywords:** canine, genetic analysis, lymphangioma, lymphangiomatosis, lymphatic malformation, minocycline, multimodal treatment, sclerotherapy

## Abstract

Lymphatic malformation is a benign congenital vascular anomaly that is rarely reported in veterinary medicine. Complete surgical excision is extremely challenging because the boundary between the abnormal and normal tissues is poorly defined, which frequently results in recurrence. A 3-year-6-month-old castrated male mixed-breed dog (Chihuahua × Miniature Dachshund) presented with recurrent lymphatic malformations in the left inguinal and peripenile regions. The lesion had previously been surgically excised at another hospital but recurred shortly after surgery. Despite long-term prednisolone therapy, the lesion gradually enlarged, resulting in gait disturbance and decreased activity. Extensive surgical excision was performed at our hospital; however, early postoperative recurrence was observed. Repeat sclerotherapy with minocycline was subsequently administered for the recurrent cystic lesions. The large cystic lesions resolved or remained controlled after treatment, and long-term suppression of lesion progression was achieved. Although small residual lesions persisted, the dog maintained good activity and gait without marked progression at 535 days postoperatively. In addition, amplicon-based targeted deep sequencing of the PIK3CA hotspot regions did not identify any pathogenic variants. This case suggests that repeated minocycline sclerotherapy may represent a potential minimally invasive treatment option for canine lymphatic malformations that are difficult to control with surgical excision alone.

## 1 Introduction

Lymphatic malformations are congenital benign vascular malformations that have been reported in humans (1). They are characterized by the proliferation of dilated abnormal lymphatic tissues that form cystic structures, resulting in various clinical manifestations (1). Reports in veterinary medicine are rare, and various terms, including “lymphangioma” and “lymphangiomatosis,” have been used interchangeably (2–6); however, the term “lymphatic malformation” is used consistently throughout this report. Although the etiology remains unclear, recent studies in humans have demonstrated a high frequency of hotspot mutations in PIK3CA within abnormal lymphatic endothelial cells, suggesting an association between these mutations and the disease (7–9). However, the genetic alterations associated with this disease have not been clarified in veterinary medicine.

The standard treatment options in humans are broadly categorized into surgical excision, sclerotherapy, and medical therapy. Surgical excision is considered potentially curative (10); however, complete resection is often difficult because the boundary between the lesion and normal tissue is poorly demarcated (1), which increases the risk of functional impairment and recurrence (11, 12). Sclerotherapy is a standard treatment modality alongside surgical excision and is widely used as an initial treatment or for recurrent lesions after surgery because of its minimally invasive nature and practicality. Reported sclerosants include OK-432, bleomycin, and tetracycline antibiotics (13).

However, there are limited reports describing the treatment of this disease in veterinary medicine. Most previously reported cases were treated surgically (2, 3). However, because complete excision is difficult owing to indistinct lesion margins, long-term recurrence-free outcomes following surgical excision alone have been reported in only a single case, in which the lesions were confined to the spleen (4).

To our knowledge, only one veterinary study has described the use of sclerotherapy for lymphatic malformations (6). However, because sclerotherapy was combined with surgical excision, electrochemotherapy, and radiation therapy, its independent therapeutic effects cannot be determined. Therefore, the efficacy of sclerotherapy for veterinary lymphatic malformations remains unclear.

In this report, we describe a canine case of lymphatic malformation that was difficult to control with surgical excision and prednisolone therapy and was subsequently managed with minocycline sclerotherapy, resulting in long-term local disease control.

## 2 Case

### 2.1 Case presentation

A castrated male mixed-breed dog (Chihuahua × Miniature Dachshund), aged 3 years and 6 months, was referred to our hospital. Multiple vesicular masses had been noted on the left side of the penis from a young age, and extensive surgical excision had previously been performed at another hospital. However, local recurrence developed shortly after surgery, and histopathological examination of the resected tissue led to a diagnosis of lymphatic malformation. Subsequently, medical management with prednisolone (0.5–2.0 mg/kg/day) was continued for approximately 3 years, but the lesions gradually enlarged, resulting in gait disturbance and decreased activity, prompting referral to our hospital.

At initial presentation, the dog was reluctant to walk voluntarily and remained in a sitting position. Physical examination revealed generalized skin thinning, a coarse hair coat, and alopecia, which were considered secondary to long-term prednisolone administration. A soft, fluctuant, multilobulated mass measuring approximately 100 × 60 × 40 mm was present in the left inguinal region (Figure 1). No erythema, pruritus, or pain was observed at the lesions. No clinically relevant abnormalities were detected on routine physical examinations, complete blood count, or serum biochemical analysis. Ultrasonography revealed multiple cystic structures within the mass (Figure 1). Doppler interrogation was performed when differentiation between cystic lesions and vascular structures was difficult and confirmed the absence of internal blood flow; Doppler images were not routinely recorded. Preoperative images were acquired using a 16-slice CT scanner (Bright Speed Elite; GE Healthcare) with single-energy CT, whereas postoperative images were acquired using a 64-detector CT scanner (Revolution Frontier; GE Healthcare) with dual-energy CT. Iohexol (Omnipaque 300, GE Healthcare) was administered via the cephalic vein at a dose of 2.0 mL/kg (600 mgI/kg) over 20 s, followed by a saline flush (0.5 mL/kg over 5 s). Equilibrium phase images were obtained 3 min after contrast injection. Preoperative CT images were acquired at 120 kV, whereas postoperative CT images were acquired using fast kV switching (80/140 kV). The slice thickness was 0.625 mm. Contrast-enhanced CT revealed multiple variably sized cystic spaces separated by internal septa within the mass, with the largest cystic lesion measuring 30 × 28 × 32 mm. The cystic contents were hypoattenuating and showed no contrast enhancement, whereas the septa showed contrast enhancement, which was most pronounced in the equilibrium phase. The mass extended subcutaneously along the abdominal wall from the left side of the penis to the left inguinal region, encased the os penis, and was in contact with the urethra (Figure 2A).

**Figure 1:**
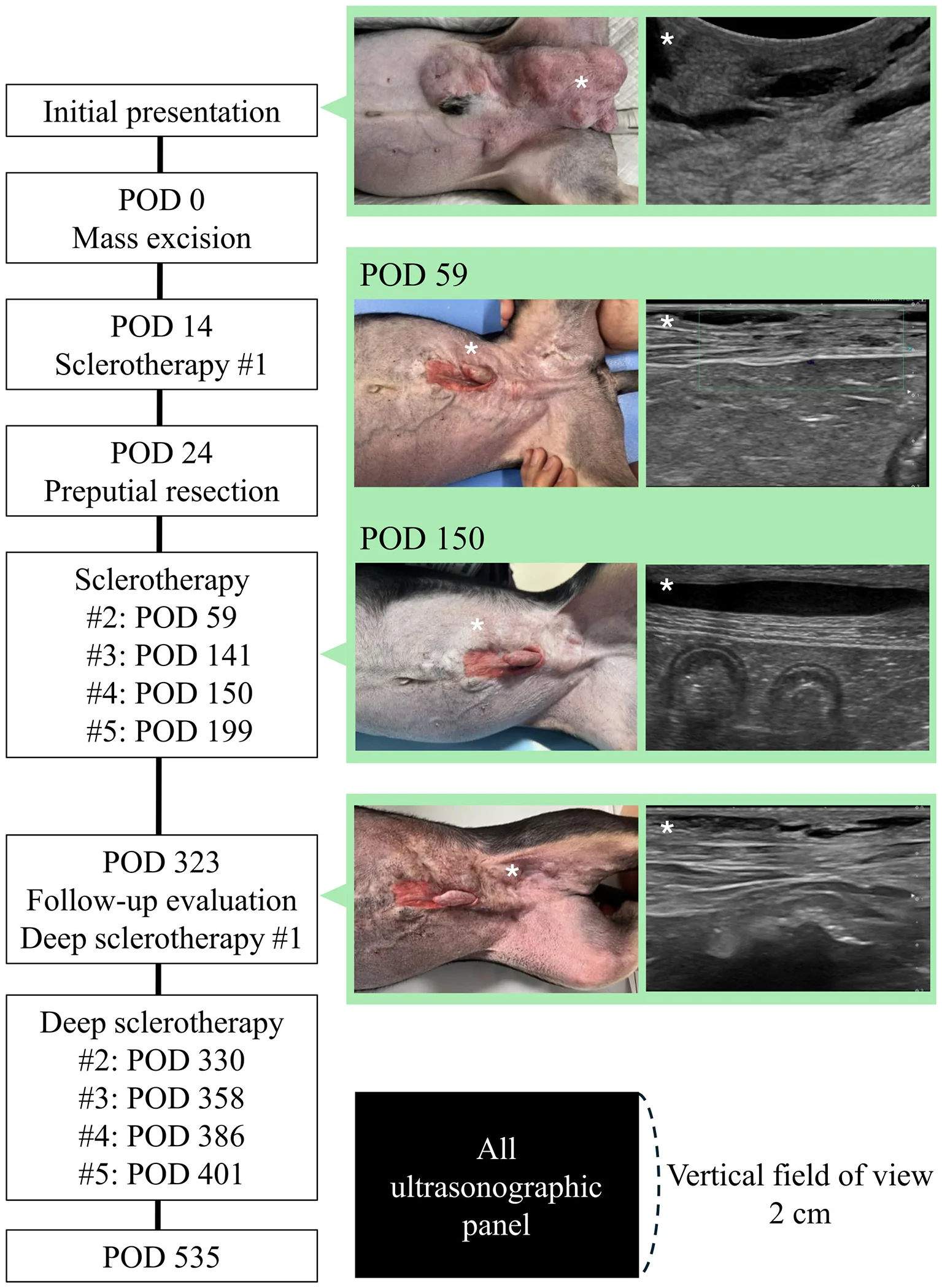
Treatment timeline and serial gross and ultrasonographic findings from initial presentation to postoperative day 535. In each gross image, the asterisk (*) indicates the approximate anatomical region corresponding to the adjacent ultrasonographic image. The ultrasonographic images were obtained directly over the mass in the left inguinal region at initial presentation, on the left side of the penis on PODs 59 and 150, and in the left inguinal region on POD 323. All ultrasonographic images were obtained in a longitudinal orientation. The ultrasonographic panels were standardized using the original depth scale so that the vertical field of view represents 2 cm. At initial presentation, a mass containing multiloculated cysts was observed subcutaneously from the peripenile region to the left inguinal region. Following surgical excision, subcutaneous cystic lesions and cutaneous nodules developed diffusely, predominantly within the same region. Sclerotherapy was performed on postoperative days (PODs) 14, 59, 141, 150, and 199. After the fifth session (POD 199), the lesions showed no marked progression over the following 124 days, until POD 323. Deep sclerotherapy was subsequently performed on PODs 323, 330, 358, 386, and 401. At the final assessment on POD 535, multiple cutaneous nodules and small subcutaneous cysts remained; however, no marked enlargement of the lesions was observed.

**Figure 2:**
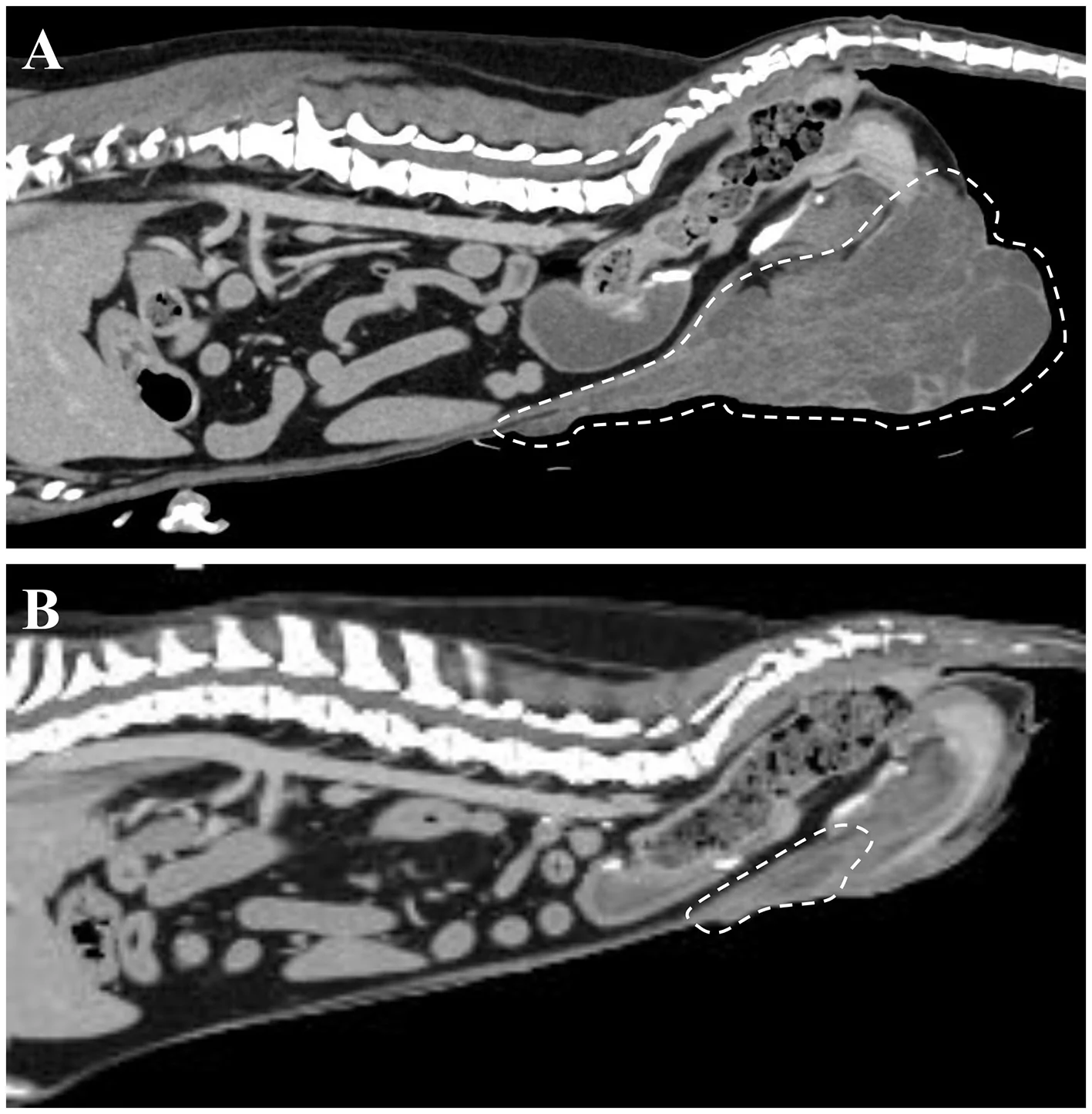
Contrast-enhanced sagittal reformatted CT images in the equilibrium phase. **(A)** CT image obtained at the initial presentation, showing a multiloculated cystic mass extending subcutaneously from the peripenile region to the left inguinal region. The mass measured approximately 100 × 60 × 40 mm, and the largest cystic lesion measured 30 × 28 × 32 mm. The lesion consisted of multiple hypoattenuating cystic spaces of various sizes separated by contrast-enhancing septa. **(B)** CT image obtained on postoperative day 323, showing marked regression of the large cystic lesions following repeated minocycline sclerotherapy. Small residual cystic lesions remained scattered in the left cranial abdomen and from the peripenile region to the left inguinal region. The largest cutaneous nodule measured approximately 11 × 14 × 7 mm, and the largest residual cystic lesion measured 7 × 2.5 × 3.5 mm. The white dashed lines delineate the overall extent of the lesion in each panel.

Surgical excision was performed after obtaining the owner's consent. A lateral surgical margin approximately 10 mm from the gross lesion was secured, and part of the external abdominal oblique muscle infiltrated by the cystic lesions was also resected. Although cystic lesions were observed in the distal penis, the penis was preserved according to the owner's preference.

The resected mass was submitted to an external laboratory (NORTH LaB, Hokkaido, Japan) for histopathological examination. The lesion extended diffusely from the deep dermis to the subcutaneous tissue with poorly defined borders and consisted of multiple irregularly dilated vascular structures of varying sizes (Supplementary Figure S1). No malignant features were identified, and the lesion was diagnosed as a recurrent lymphatic malformation.

Fourteen days after surgery, ultrasonography revealed multiple cystic lesions in the subcutaneous tissues of the left cranial abdomen and around the penis. Based on blood examination and cytologic examination of the cystic fluid, which revealed no bacteria or cytologic evidence of infection, infection was considered unlikely. Bacterial culture was therefore not performed. Recurrence of the lymphatic malformation was suspected, and after obtaining informed consent from the owner, sclerotherapy with minocycline as a sclerosant was performed as an additional treatment. Minocycline was used at a final concentration of 15 mg/mL and mixed with 2% lidocaine to reduce pain. Target cysts were selected based on ultrasonographic findings, with priority given to lesions with the largest diameters. Each lesion was punctured using a 23-gauge needle, the cystic fluid was aspirated, and enough of the solution was injected to fill the cystic cavity and left in place without subsequent aspiration. One to three lesions were treated per session. The total dose was limited to a maximum of 10 mg/kg, and this maximum dose was administered at each treatment session.

On postoperative day 24, urine leakage caused by dehiscence of the preputial mucosal suture line was observed in the subcutaneous tissue on the left side of the penis. Revision surgery and partial preputial resection were performed.

Follow-up examinations, including ultrasonography, were performed approximately once a month. The cystic lesions and palpable cutaneous nodules with induration gradually increased, mainly around the penis and left inguinal region. Additional sclerotherapy sessions were performed on postoperative days 59, 141, 150, and 199 for cysts measuring ≥10 mm in maximal diameter that were amenable to puncture, resulting in a total of four additional treatments. The dog was re-examined earlier than scheduled on postoperative day 150, at which time a separate cystic lesion meeting the treatment criteria was identified and treated.

After postoperative day 199, the remaining lesions consisted mainly of small cysts < 10 mm in maximal diameter, with no marked increase in the number or size of cutaneous nodules. As the general condition and gait remained favorable, the dog was subsequently monitored with prednisolone therapy alone for approximately 4 months.

On postoperative day 323, multiple cutaneous nodules extending from the penis to the left inguinal region were observed, with the largest measuring approximately 11 × 14 × 7 mm (Figure 1). The dog's activity and gait were maintained. Ultrasonography and contrast-enhanced CT revealed small scattered cystic lesions in the left cranial abdomen and extending from around the penis to the left inguinal region, with the largest residual cystic lesion measuring 7 × 2.5 × 3.5 mm (Figures 1, 2B).

To reduce the number of cutaneous nodules, small, deep cystic lesions adjacent to the nodules were additionally targeted for sclerotherapy. The other aspects of the treatment protocol remained unchanged. Deep sclerotherapy was administered on postoperative days 323, 330, 358, 386, and 401. On postoperative day 444, the cutaneous nodules showed marked temporary regression (Figure 3); however, they recurred shortly thereafter. The dog was monitored, and prednisolone was gradually tapered. At 535 days postoperatively, some cutaneous nodules and small cysts remained; however, no marked progression or enlargement was observed, and the dog's activity and gait were well maintained.

**Figure 3:**
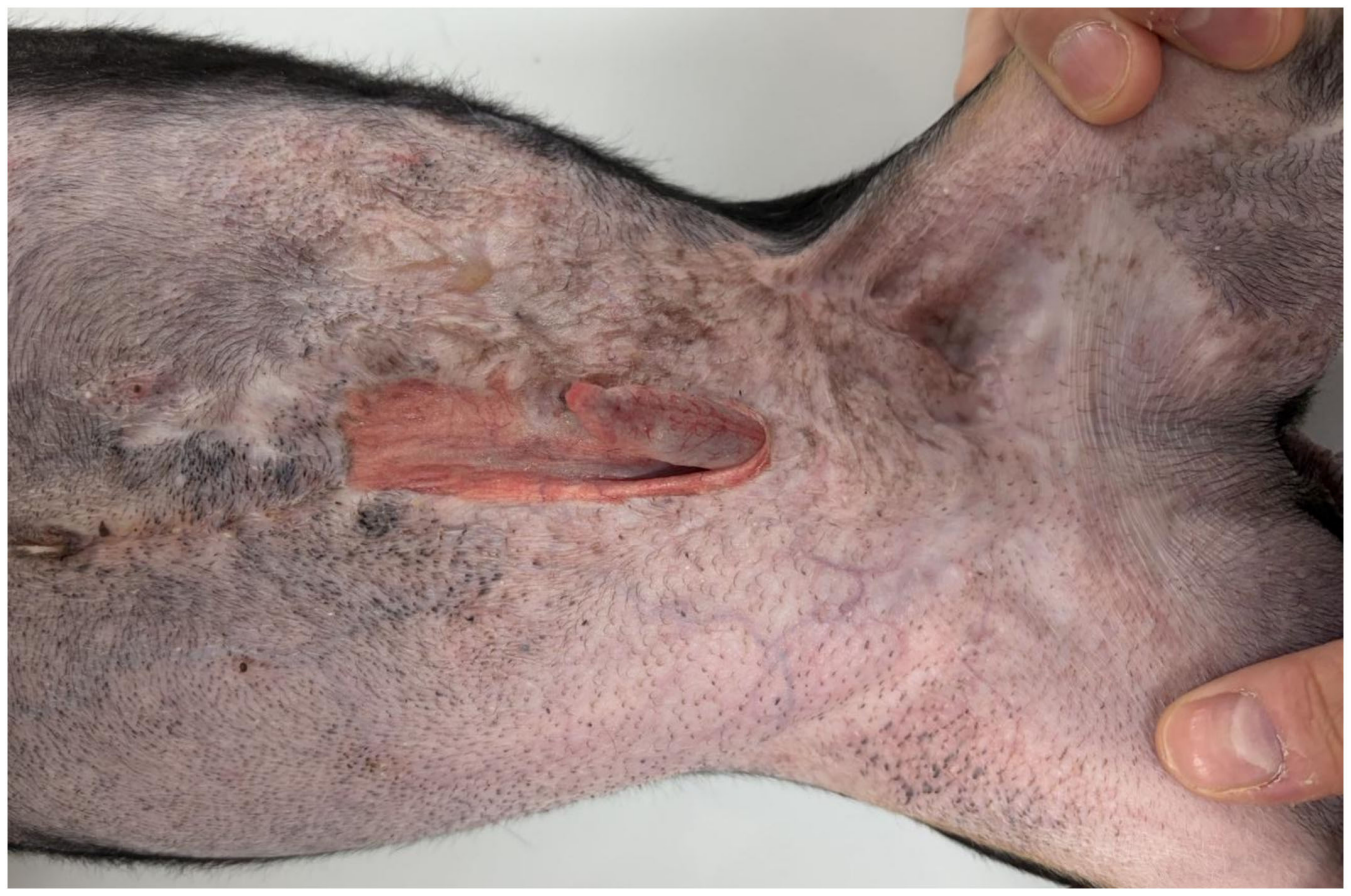
Gross appearance on postoperative day 444. After completion of deep sclerotherapy, the cutaneous nodules markedly regressed; however, they recurred shortly thereafter.

Adverse events associated with sclerotherapy included brief vocalization during injection in several treatment sessions and decreased activity on the day of treatment, as reported by the owner. Pain and discomfort were assessed after each procedure, and because the observed signs were mild and transient, no additional analgesic medications were administered. Localized skin necrosis at the injection site developed after each of three treatment sessions and was observed on postoperative day 73, 150, and 416; however, all lesions healed uneventfully by secondary intention (Figure 4).

**Figure 4:**
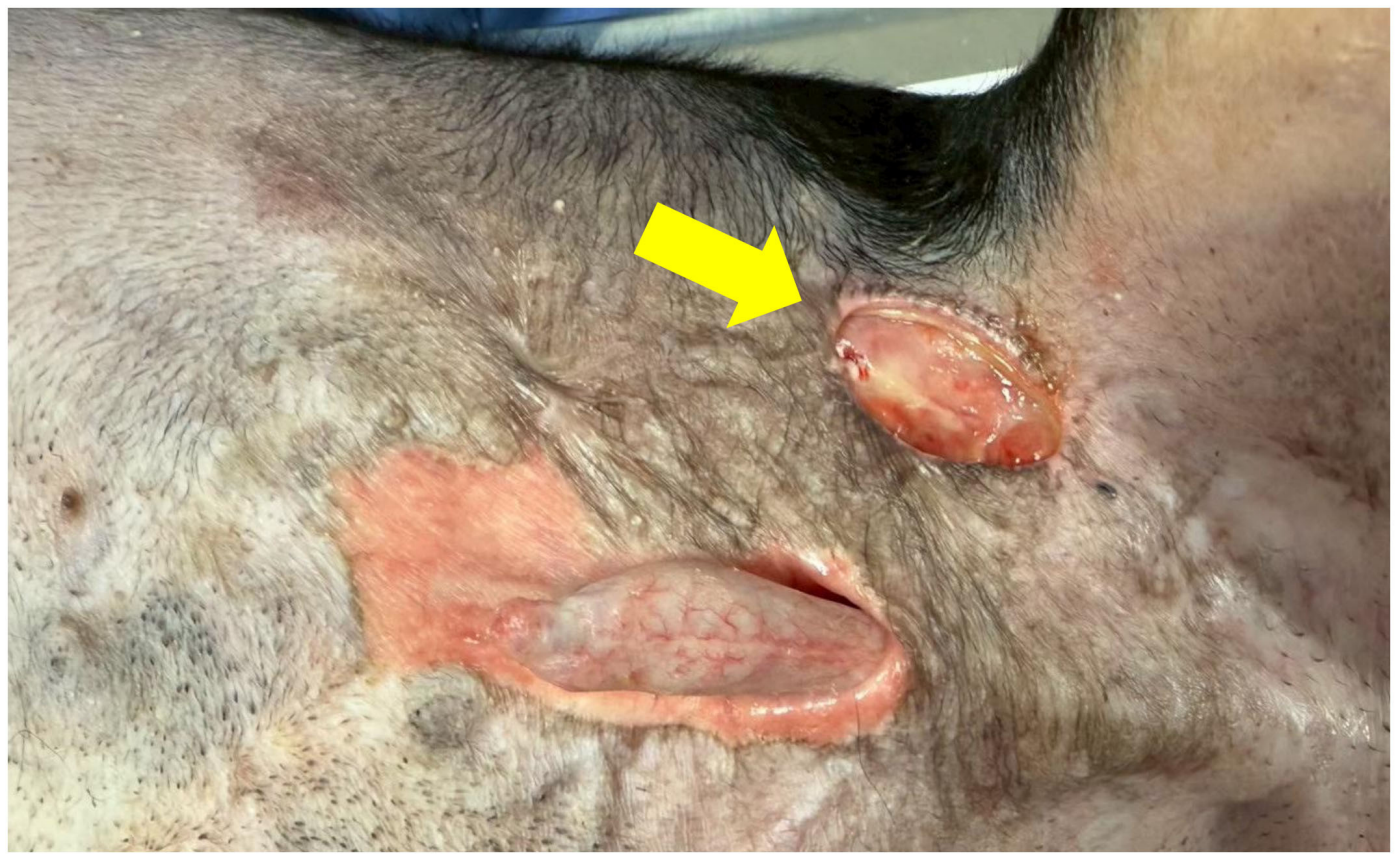
Skin sloughing (arrow) was caused by an adverse effect of sclerotherapy on postoperative day 150. Localized skin necrosis of a similar size to that shown in the figure developed after each of three treatment sessions; however, all lesions healed uneventfully by secondary intention.

### 2.2 Genetic analysis

For PIK3CA analysis, DNA was extracted from tissue samples collected at the time of lesion resection using a QIAamp DNA Mini Kit (Qiagen, Hilden, Germany). Polymerase chain reaction (PCR) and amplicon-based targeted deep sequencing were performed by Bioengineering Lab. Co., Ltd. (Kanagawa, Japan).

PIK3CA gene analysis was performed using a two-step PCR approach targeting two long-range amplicons encompassing exons 9–15 and exons 16–20, respectively. The primer sequences are listed in Supplementary Table S1. PCR was performed using PrimeSTAR GXL DNA Polymerase (Takara Bio, Shiga, Japan): initial denaturation at 98 °C for 3 min; 30 cycles at 98 °C for 10 s, 60 °C for 15 s, and 68 °C for 8 min (exons 9–15) or 6 min (exons 16–20); final extension at 68 °C for 5 min; and eight second-round PCR cycles. Revio sequencing (Pacific Biosciences) generated circular consensus reads, which were quality-filtered (average score ≥20), primer-trimmed, and mapped to the canine reference genome (NC_051838.1) using pbmm2. Single-nucleotide variants and indels were called using DeepVariant (v1.6.0). Because lymphatic malformations are often driven by low-frequency mosaic mutations (9), we subsequently performed a highly sensitive, full-depth allele counting approach. Base pileups were generated using SAMtools mpileup without depth capping to comprehensively screen for low variant allele frequency (VAF) candidates (VAF ≥ 1%, read depth ≥ 1000 × ) across the targeted amplicons. Furthermore, to evaluate the presence of known pathogenic variants in humans, we directly quantified reference and alternative read counts at specific loci orthologous to human PIK3CA hotspot mutations (E542, E545, Q546, and H1047; Supplementary Figure S2) (8, 9). To exclude false-positive calls caused by alignment artifacts, low-level single-nucleotide variant candidates were assessed for their proximity to indels. All detected candidates were subsequently confirmed using Integrative Genomics Viewer.

Long-range PCR successfully generated two amplicons covering PIK3CA exons 9–15 and exons 16–20. Reanalysis of the sequencing data identified five low-level single-nucleotide variant candidates (VAF, 1–10%). However, all candidates were located within 4 bp of known indels and were therefore considered likely alignment artifacts. No known pathogenic PIK3CA variants were detected in any of the analyzed regions. Analysis of the loci orthologous to the human PIK3CA hotspot mutations E542, E545, Q546, and H1047 revealed no known pathogenic hotspot variants. These regions were covered at an approximate read depth of 44,000 × and variant-supporting reads were detected only at background noise levels (maximum VAF, 0.0068%).

## 3 Discussion

In the present case, repeated minocycline sclerotherapy provided a minimally invasive approach that achieved long-term disease control of a lymphatic malformation that was resistant to prednisolone therapy and recurred shortly after surgical excision.

In dogs, lymphatic malformations have been reported to cause a variety of clinical signs owing to physical compression by cystic lesions and lymphatic fluid leakage (2–6). Reported complications include ureteral obstruction (3), as well as gastrointestinal compression (6). In this case, the marked improvement in activity and gait disturbance following surgical excision suggested that these clinical signs were primarily caused by the physical effects of the cystic lesions.

CT findings in the present case were characterized by multiple variably sized cystic spaces, contrast-enhancing septa separating these spaces, and non-enhancing cystic contents. Similar features have been reported previously (3, 5, 6). However, such findings have also been reported in canine abscesses and cystic neoplasms (14, 15) and are therefore not specific to lymphatic malformations.

Gross distinction between abnormal lymphatic tissue and normal tissue is difficult in lymphatic malformations (1). Accordingly, although the lesion in this case was resected with a 10-mm lateral margin from the gross lesion and included the underlying muscle layer, recurrence developed shortly after surgery. Complete excision may require more extensive resection, but functional impairment and reconstruction may limit feasibility. In humans, sclerotherapy has been reported to be useful treatment for postoperative recurrence and when surgical excision is difficult (12, 16). However, complete resolution is not always achieved, and its effects on small cysts are limited (12, 16). In the present case, lesions were classified using the 10-mm cutoff reported in human medicine (17). Cysts measuring ≥10 mm disappeared, whereas smaller cysts persisted.

The cutaneous nodules observed in this case resembled superficial lymphatic malformations reported in humans, in which clusters of cystic lesions were present within the adjacent deep subcutaneous tissue (18). Based on previous reports, sclerotherapy was additionally applied to these deep cystic lesions (19), resulting in a marked but temporary regression of the cutaneous nodules. These findings suggest that the cutaneous nodules observed in this case were secondary lesions associated with underlying deep cysts (20).

Minocycline, used as a sclerosant, is thought to induce endothelial cell injury because of its strong acidity (pH 2.0–3.0) and cytotoxicity, thereby suppressing the reaccumulation of cystic fluid through fibrosis of the cyst wall (21). In humans, its safety and efficacy have been reported for postoperative lymphatic leakage and hepatic and renal cysts (21–23). In the present case, treatment with minocycline for lymphatic malformations prevented marked enlargement of the cystic lesions during long-term follow-up. These findings suggest that minocycline may be a useful sclerosant for the management of lymphatic malformations.

In humans, intralesional doxycycline, another tetracycline antibiotic, has been administered at doses of up to 20 mg/kg per session (24), approximately 4.5 times the recommended systemic daily antimicrobial dose (25). Major and minor complication rates of 2% and 10%, respectively, were reported, and the ability to inject relatively large amounts of doxycycline without toxicity was described as a major advantage (24). However, whether this dosing approach can be directly extrapolated to dogs has not been sufficiently evaluated. Therefore, in the present case, the maximum total dose of minocycline was set at 10 mg/kg, based on the standard antimicrobial dose in dogs.

In humans, lidocaine has been added to tetracycline-based sclerosants to reduce injection-related pain (26), and the analgesic benefit of lidocaine during sclerotherapy has been demonstrated in a randomized controlled study (27). Based on these findings, minocycline was mixed with lidocaine in the present case. Although no severe pain responses were observed, occasional vocalization immediately after injection and transiently decreased activity suggested that mild discomfort was not completely prevented. This finding is consistent with previous reports indicating that lidocaine may reduce but does not eliminate treatment-related pain. In human medicine, the concomitant use of acetaminophen and nonsteroidal anti-inflammatory drugs has been reported for pain management (28), and these medications may represent potential options for dogs. Localized skin necrosis is a rare complication in humans (29). Because tetracycline antibiotics possess cytotoxic properties, excessive tissue injury and subsequent necrosis may occur, depending on the drug concentration and treatment interval. In addition, long-term administration of prednisolone in this case resulted in marked skin thinning, which may have partially contributed to the development of skin necrosis.

No pathogenic PIK3CA hotspot variants were detected; low-level non-hotspot candidates were considered likely alignment artifacts. One possible explanation for the absence of pathogenic mutations is that genetic abnormalities other than PIK3CA may contribute to disease development. Indeed, in humans, cases harboring NRAS mutations (30) and those lacking detectable PIK3CA mutations have been reported (8). Alternatively, the sample may have contained insufficient abnormal lymphatic endothelial cells, causing any pathogenic variants to be diluted below the detection threshold by surrounding normal-cell DNA. In contrast, the long-range PCR method used in this study enabled stable amplification of PIK3CA exons 9–15 and exons 16–20, allowing for high-depth analysis of each hotspot region. Therefore, this method may be useful for PIK3CA hotspot analysis in dogs and could provide a basis for future molecular genetic investigations of lymphatic malformations.

Several limitations should be considered before concluding that sclerotherapy is effective for lymphatic malformations based on this case alone. First, this report describes only a single case; therefore, the efficacy and safety of the treatment should be interpreted with caution. Further accumulation of such cases is needed. Second, dedicated lymphatic imaging techniques, such as CT lymphangiography, and magnetic resonance imaging were not performed. Therefore, the full extent of the lesions may not have been completely assessed. Third, because surgical excision substantially reduces the volume of abnormal lymphatic tissue, it remains uncertain whether large preoperative cystic lesions would have recurred in the absence of sclerotherapy. Fourth, the maximum minocycline dose of 10 mg/kg was based on the systemic antimicrobial dose in dogs, and the safety of intralesional minocycline administration in dogs has not been established. Fifth, changes in the number and size of individual cystic lesions were not systematically quantified during follow-up, apart from the paired measurement of the largest cystic lesion on the preoperative and POD 323 CT studies. Because numerous cystic lesions of varying sizes were distributed over a broad area, consistent longitudinal tracking of the same lesions and quantitative assessment of the overall lesion burden by ultrasonography were difficult. Therefore, treatment response was evaluated primarily based on clinical findings and serial imaging examinations, which may have limited the objective assessment of lesion progression and treatment efficacy. Nevertheless, long-term clinical stability was maintained throughout the follow-up period, and no recurrence of large cystic lesions was observed, suggesting that the treatment contributed to sustained disease control.

In conclusion, minocycline sclerotherapy may achieve long-term disease control in lymphatic malformations that are refractory to surgical excision alone.

## Data Availability

The PIK3CA targeted deep-sequencing data presented in this study are deposited in the DDBJ Sequence Read Archive, accession number DRA029611 (https://ddbj.nig.ac.jp/resource/sra-submission/DRA029611). The data are linked to BioProject accession number PRJDB42947 and BioSample accession number SAMD01956689.
