# Supplementary material for: Case report: Long-term local control of recurrent lymphatic malformations using minocycline sclerotherapy following surgical resection in a dog
Source: Front Vet Sci. 2026 Sep 17;13:1910356. doi: 10.3389/fvets.2026.1910356 (PMC13627013; doi:10.3389/fvets.2026.1910356)
Supplement: Supplementary file 1 [file Data_Sheet_1.docx]

Supplementary Material

## 1.1 Supplementary Figure S1

**Figure S1. Histopathological findings of the recurrent lymphatic malformation.**

Histopathological examination revealed multiple irregularly dilated lymphatic channels of varying sizes within the connective tissue. The channels were lined by a single layer of flattened endothelial cells and contained fluid without erythrocytes. Hematoxylin and eosin staining. Scale bar = 100 μm.

## 1.2 Supplementary Figure S2

**Figure S2. Genomic mapping of targeted PIK3CA amplicons and corresponding hotspot mutations.**

To determine the exact genomic coordinates corresponding to the canonical human PIK3CA hotspot sites, the reference dog PIK3CA mRNA sequence was splice-aligned to the genomic reference sequence using minimap2. Sequence analysis confirmed that the amino acid residues corresponding to the canonical human PIK3CA hotspot sites (E542, E545, Q546, and H1047) are perfectly conserved in the canine PIK3CA protein. Genomic coordinates shown in the figure represent relative positions within the canine PIK3CA gene.
