# Supplementary material for: Case report: Long-term local control of recurrent lymphatic malformations using minocycline sclerotherapy following surgical resection in a dog
Source: Front Vet Sci. 2026 Sep 17;13:1910356. doi: 10.3389/fvets.2026.1910356 (PMC13627013; doi:10.3389/fvets.2026.1910356)
Supplement: Supplementary file 2 [file Table_1.docx]

Supplementary Material

## 1.1 Supplementary Table

**Table S1. Primer sequences used for long-range PCR amplification of canine PIK3CA.**

| Amplicon | Target region | Primer | Sequence 5'→3' |
| --- | --- | --- | --- |
| 1 | PIK3CA exon 9–15 | Forward | GATCACTTGTGAATCCAGGGAAAAATG |
|  | PIK3CA exon 9–15 | Reverse | ATTGGGGTTAACAGAATTCAGGGCC |
| 2 | PIK3CA exon 16–20 | Forward | GGCCCTGAATTCTGTTAACCCCAAT |
|  | PIK3CA exon 16–20 | Reverse | CACCAAACTTCAGTTTCCTTGGACC |
